# Exploring English and Swedish General Practitioners’ Behavioral Intentions to Use Telemedicine: Comparative Study

**DOI:** 10.2196/73609

**Published:** 2026-03-20

**Authors:** Michael Harris, Gordon Taylor, Miriam Pikkemaat, Hans Thulesius, Veronica Milos Nymberg

**Affiliations:** 1 College of Medicine and Health, University of Exeter, Exeter, United Kingdom; 2 Bern Institute of Primary Health Care (BIHAM), University of Bern, Bern, Switzerland; 3 Center for Primary Health Care Research, Department of Clinical Sciences Malmö, Lund University, Jan Waldenströms gata 35, Malmö, 20213, Sweden, 46 0767700240; 4 University Clinic Primary Care, Skåne University Hospital, Region Skåne, Sweden

**Keywords:** telemedicine, general practitioners, GP, theory of planned behavior, behavioral intentions, attitudes, perceived behavioral control, subjective norms

## Abstract

**Background:**

Although telemedicine grew rapidly during the COVID-19 pandemic, instruments to assess general practitioners’ (GPs) attitudes and behavioral intentions to use it are scarce. In Sweden, the Physicians’ Attitudes and Intentions to use Telemedicine (PAIT) questionnaire was developed from the “theory of planned behavior” in 2019 and translated into English in 2022.

**Objective:**

The aim of this study was to explore similarities and differences between behavioral intentions and predictors of intentions to use telemedicine among GPs in England and Sweden.

**Methods:**

This study compared attitudes, behavioral intentions, and self-reported use of telemedicine after the COVID-19 pandemic among 52 GPs in England and 101 GPs in Sweden. The PAIT questionnaire has 33 items with 7-point Likert scale options ranging from “strongly disagree” to “strongly agree,” examining 3 predictors of intentions: attitudes (12 items), subjective norms (6 items), perceived behavioral control (9 items), and “intentions” (6 items) to use telemedicine; 22 items assess use of telemedicine tools, general questions about telemedicine, training experience, free-text comments, and demographic and background questions.

**Results:**

Both English and Swedish GPs reported little training and low use of telemedicine after the COVID-19 pandemic. Swedish GPs had significantly higher mean scores for intentions to use telemedicine in daily practice compared with English GPs. More positive attitudes and higher perceived behavioral control were significantly associated with higher behavioral intention scores in both English and Swedish GPs.

**Conclusions:**

While our results are exploratory due to sample size constraints, these findings provide insights into the similarities and differences between English and Swedish GPs regarding telemedicine adoption—attitudes, behavioral intentions, and self-reported use of telemedicine assessed by the PAIT questionnaire—which proved useful for cross-country comparisons and could be used for further international studies.

## Introduction

### Background

Telemedicine has developed at a rapid pace and increased in use worldwide in recent years [1,2], partly due to social distancing policies introduced during the COVID-19 pandemic [3-6]. Current telemedicine technologies are available at a low cost, accessible to those living in rural areas, and they have the potential to decrease the workload for general practitioners (GPs) [7,8].

Telemedicine is defined by the United Kingdom’s National Health Service as “The use of telecommunication and information technology providing remote health assessments and therapeutic interventions” [9]. Another definition is “health care service delivered by health care providers in a patient-centered manner, from a geographical distance using ICT (information and communication technology)” [10]. Telehealth is a broader term encompassing the integration of telecommunication in the practice, promotion, and management of health care. Methods of communication in telemedicine include video consultations, telephone calls, emails, as well as mobile apps to monitor chronic diseases (mobile health), digital diagnostic algorithms, triage, and web guideline support for treatments [7,8,11,12].

While studies in the United Kingdom have shown that GPs have a positive attitude toward telemedicine, some have criticized the lack of IT integration with existing medical systems and reported a lack of telemedicine experience, especially before the COVID-19 pandemic [3,7]. Surveys in the United States and Sweden have shown similar conclusions: positive attitudes toward telemedicine but little use of it [11,13-16]. The main obstacles for use have been reported to be a lack of experience and understanding of the use of these communication methods and technologies [13,16]. The increased use of telemedicine in health care [2,6] has raised concerns about both GP workload increase and decrease [13], with Salisbury et al [17] warning of an increase in GPs’ workload when integrating telemedicine as a means of digital consultation before face-to-face consultation. Workload is only decreased if at least half of digital consultations are nonurgent matters, and it depends on the demand and requirements of the consultations [17]. There has been a call for research on telehealth and telemedicine to evaluate how they could improve medical consultations and disease monitoring [1,14]. In this study, telemedicine refers to remote clinical consultations between patients and clinicians, such as by telephone, video, chat, or secure email. It does not include remote monitoring devices, digital diagnostics, or triage algorithms.

### Theoretical Framework

Over the past few decades, several theories have emerged to explain end-user acceptance and adoption of new technologies [10,18-22]. One of the most commonly applied frameworks for understanding telemedicine adoption among health care staff is the theory of planned behavior (TPB).

The TPB explains how behavioral intentions influence the likelihood of performing an action. According to the theory, these intentions are shaped by an individual’s attitudes toward the behavior, subjective norms—defined as the individual’s perception of social expectations regarding behavior change—and perceived behavioral control, which reflects the individual’s belief in their ability to perform the behavior [23].

The TPB is widely recognized for its practical applicability and predictive validity. A validated manual is available for implementing the TPB, enabling the development of questionnaires designed to assess behavioral intentions [23].

This study aims to build upon the Swedish study by Pikkemaat et al [11], conducted in 2019 before the COVID-19 pandemic. That study examined physicians’ attitudes and intentions toward using telemedicine, using a newly designed survey instrument based on the TPB. The Swedish-language instrument, known as the Physicians’ Attitudes and Intentions to use Telemedicine (PAIT) survey, was distributed to 820 potential respondents across 160 health care centers in 2 southern Swedish regions, yielding 198 (24.1%) responses. The study found high levels of intention and positive attitudes toward using telemedicine, with perceived behavioral control emerging as the strongest predictor of telemedicine adoption. Given that the COVID-19 pandemic accelerated the use of telemedicine, the study suggested that intentions to use digital tools in primary care may have increased since 2019, warranting further exploration across different contexts.

To date, no studies have examined the intentions of English GPs to adopt telemedicine. Building on insights from the Swedish study conducted before the COVID-19 pandemic [11] and the assumption that telemedicine adoption may have shifted in primary care following the pandemic, we decided to collect new survey data from Sweden and compare it with data from England, both within the primary care setting.

The objective of this study was to explore and compare the intentions of English and Swedish GPs to use telemedicine using the TPB-validated questionnaire. Additionally, the study aimed to assess how attitudes, subjective norms, and perceived behavioral control influence the intentions of GPs in both countries to adopt telemedicine.

## Methods

### Survey Design

The PAIT survey instrument was originally designed and written in Swedish by Pikkemaat et al [11], based on the manual from the University of London Institutional Repository for creating questionnaires using the TPB, constructed by Francis et al [24]. The PAIT survey includes questions about experiences with digital contact methods, chronic disease monitoring with digital tools, and participant demographics. Digital contact methods encompass the various telemedicine approaches GPs might use to communicate with patients, such as telephone, online chat, video consultation, and email.

The original PAIT-Swedish survey instrument also included questions regarding experiences with artificial intelligence (AI) in telemedicine, focusing on diagnostic algorithms, triage, and support for treatment guidelines. However, as responses from the Swedish survey indicated very low use and experience of AI technologies among GPs [11], these questions were excluded from the English survey.

### Translation and Validation Process

The PAIT survey was translated into English by a Swedish-English bilingual master’s student, with minor cultural adaptations to suit the UK health care context. To ensure linguistic equivalence and conceptual accuracy, the English version underwent back-translation into Swedish, followed by a detailed comparison with the original Swedish version. This process was overseen by the research team, ensuring that no essential meaning was lost during translation.

The newly adapted English-language version, named “Behavioural Intentions towards Telemedicine” (BIT), was designed to yield scores comparable to both the pre–COVID-19 pandemic PAIT-Swedish survey [11] and the results from a repeat PAIT-Swedish survey conducted following the COVID-19 pandemic (Multimedia Appendix 1).

### Validation and Reliability Testing

The translated BIT survey was validated following established guidelines, including the Checklist for Reporting Results of Internet E-Surveys (CHERRIES), as recommended by the *Journal of Medical Internet Research* [25] (Checklist 1). The PAIT-Swedish version had previously undergone rigorous testing for construct validity and reliability using the Churchill Paradigm and COSMIN (Consensus-based Standards for the Selection of Health Measurement Instruments) guidelines. This involved a pilot test with 24 participants and evaluation by 5 proficient physicians in telemedicine to ensure the questionnaire accurately captured the research questions.

Internal consistency was assessed using Cronbach α, with items scoring below an acceptable threshold removed to improve the instrument’s reliability. The final version demonstrated Cronbach α scores ranging between 0.6 and 0.95. Temporal stability was further tested by administering the questionnaire to the same 24 respondents after 2 weeks, with 12 respondents completing the retest. The Pearson correlation coefficient for test-retest reliability was 0.63, prompting minor adjustments to question structures for the final version.

### Survey Distribution

The anonymous web-based BIT survey was distributed to GPs located in southwest England between March and May 2023. We contacted GPs through email and social media (Twitter and Facebook), as well as through the Royal College of General Practitioners, the British Medical Journal, GP magazine, Pulse magazine, Local Medical Committees, “GP online,” “eLearning,” “GP survival,” and Somerset GP Education. An introductory email, including the survey or a request to share it, was sent to the managers of these groups, explaining the purpose of the research. The survey was only distributed if the group managers agreed to facilitate its circulation.

The survey for Swedish GPs was distributed between March and May 2022 to managers at the same 160 primary health care centers in southern Sweden as in the 2019 survey.

### Statistics

Given the small English sample size (n=52) that limited the power of the study, analyses were exploratory. Likert scale responses were converted to numerical scores, and demographic responses were coded accordingly. Likert scale scores ranged from 1 to 7, where 1 represented “not at all,” “harmful,” “bad,” “unpleasant,” or “useless,” and 7 represented “to a great extent,” “beneficial,” “good,” “pleasant,” or “useful.”

We converted respondents’ years of practice into 3 categories (<15 years, 15‐29 years, and ≥30 years). For each respondent and topic, we calculated the mean intention scores from questions 17 and 18, covering email and/or online chat, video consultation, and digital tools for chronic disease monitoring.

We also calculated scores for the core concepts of the TPB that influence behavioral intentions. This included mean scores for subjective norms (questions 15 and 16), perceived behavioral control (questions 12 to 14), and attitudes (questions 6, 8, and 11). Self-reported current use of telemedicine included using different digital tools (email, chat, and video). Data were analyzed using SPSS (version 28.0; IBM Corp). Descriptive statistics were generated separately for English and Swedish GPs across the various outcomes. Group differences between English and Swedish GPs were tested using the 2-tailed Student *t* test.

To explore the association between predictor scores and behavioral intentions to use telemedicine among English and Swedish GPs, we performed a split-sample multiple regression analysis.

Data analysis followed a consistent approach for both the English and Swedish surveys, with one exception: in the Swedish survey, respondents could select “I don’t know” for Likert scale questions. These responses were categorized as “Not Applicable” during the analysis.

### Ethical Considerations

The English ethical review was conducted by the University of Exeter Medical School’s Research Ethics Committee. Ethics approval was granted by the review board in March 2023 (845606). The study was carried out in accordance with the Declaration of Helsinki (Fortaleza, Brazil, October 2013), the UK Policy Framework for Health and Social Care Research (2020), and the general principles of Good Clinical Practice E6 (R2). Data storage complied with UK General Data Protection Regulation requirements and was securely maintained within the University of Exeter database. The study was deemed to pose no risk and did not involve the collection of any personal, health, or sensitive data.

The Swedish Ethical Review Authority provided an advisory statement confirming that, according to the Swedish Ethical Review Act (SFS 2003:460), ethical vetting was not required for this type of study because it involved anonymous data and did not inquire about participants’ own health or other sensitive topics. The statement provided by the Swedish Ethical Review Authority in November 2018 states the following: “In accordance with current ethical and data‑protection standards, the project does not require formal ethical review, as no personal data are processed and the information utilized cannot be linked to identifiable living individuals. Should it later become evident that personal data are involved, only the processing of sensitive personal data—such as information pertaining to racial or ethnic origin, political opinions, religious or philosophical beliefs, trade union membership, or data relating to health or sexual life—would necessitate formal ethical approval.”

All participants who returned questionnaires provided informed consent to participate in the study. No monetary compensation was offered to the participants.

## Results

We received 52 responses from English GPs and 101 responses from Swedish GPs. The demographics of the respondents varied significantly between the two groups (Table 1). The mean age of English GPs was 51.2 years, compared with 47.8 years for Swedish GPs.

**Table 1.** Demographic characteristics of respondents.

Characteristics	English GPs^a^ (n=52), n (%)	Swedish GPs (n=101), n (%)
Gender		
Women	37 (71.2)	51 (50.5)
Men	15 (28.8)	48 (47.5)
Nonbinary	0 (0.0)	1 (1.0)
Training in telemedicine		
Yes	17 (32.7)	37 (36.6)
No	35 (67.3)	64 (63.4)
Practice (years)		
<15	8 (15.4)	57 (56.4)
15‐29	23 (44.2)	36 (35.6)
≥30	21 (40.4)	8 (7.9)

a GP: general practitioner.

Gender distribution also differed, among 52 English GPs, 15 (28.8%) identified as men and 37 (71.2%) as women, while among 101 Swedish GPs, 48 (47.5%) identified as men and 51 (50.5%) as women.

Regarding telemedicine training, 17 (32.7%) English GPs reported having received such training, compared with 37 (36.6%) Swedish GPs. Additionally, English GPs had more years of practice on average than their Swedish counterparts.

Swedish GPs had significantly (*P*<.001) higher intentions to use email and/or online chat, video consultations, and digital tools to monitor chronic diseases compared with their English counterparts (Table 2).

The theoretical predictors of behavior derived from the TPB revealed significant differences between English and Swedish GPs, with Swedish GPs scoring higher for both intentions and predictors of behavioral intentions to use telemedicine (Table 3).

**Table 2.** Comparison of intentions to use different telemedicine tools between English and Swedish general practitioners (GPs).

Topics	English GPs (n=52), mean (SD)	Swedish GPs (n=101), mean (SD)	*P* value
Email and/or online chat	3.6 (2.3)	4.9 (1.8)^a^	<.001
Video consultation	2.8 (1.9)^b^	4.7 (2.0)^c^	<.001
Digital tools to monitor chronic diseases	4.3 (1.9)	5.6 (1.8)^d^	<.001

a n=99.

b n=50.

c n=100.

d n=97.

**Table 3.** Distribution of mean scores for theoretical constructs derived from the theory of planned behavior predictors (subjective norms, attitudes, and perceived behavioral control) on intentions among English and Swedish general practitioners (GPs).

Constructs	English GPs (n=52), mean (SD)	Swedish GPs (n=101), mean (SD)	*P* value
Subjective norms	2.8 (1.3)	3.4 (1.4)	.03
Attitudes	4.1 (1.1)^a^	4.1 (1.1)^b^	<.001
Perceived behavioral control	3.6 (1.0)	4.4 (1.1)	<.001
Intentions	3.7 (1.6)	4.8 (1.7)^c^	<.001

a n=51.

b n=96.

c n=99.

Overall, the self-reported use of telemedicine was low among both English and Swedish GPs (Table 4). However, the mean scores for self-reported current use indicated that English GPs were significantly more likely to use email and/or online chat (*P*=.006). There were no significant differences between the two groups regarding the use of video consultations (*P*=.76) or digital tools for monitoring chronic diseases (*P*=.73).

For both English and Swedish GPs, significant associations were observed between attitudes, perceived behavioral control, and intentions to use telemedicine (Table 5). The model had a higher explanatory power for the English GPs (*R*^2^=0.664) than for the Swedish GP (*R*^2^=0.258). However, given the modest sample sizes, particularly in the English cohort, the multivariable regression analyses should be interpreted cautiously as they may be underpowered to detect smaller effect sizes.

**Table 4.** Self-reported current use of email and/or online chat, video consultations, and digital tools for chronic disease monitoring among English and Swedish general practitioners (GPs).

Tools	English GPs (n=52), mean (SD)	Swedish GPs (n=101), mean (SD)	*P* value
Email and/or online chat	2.2 (1.2)	1.7 (1.0)	.006
Video consultation	2.0 (1.5)	1.9 (1.3)^a^	.76
Digital tools to monitor chronic diseases	2.2 (1.7)^b^	2.1 (1.6)^c^	.73

a n=96.

b n=50.

c n=98.

**Table 5.** Split sample multiple regression analysis with intention scores from English and Swedish general practitioners (GPs) as dependent variables.

Predictors^a^	Intentions of English GPs^b^			Intentions of Swedish GPs^c^		
	U-B^d^ (95% CI^e^)		*P* value	U-B (95% CI)		*P* value
Subjective norms	0.082 (−0.139 to 0.303)		.46	0.042 (−0.174 to 0.258)		.70
Attitudes	0.783 (0.453 to 1.113)		<.001	0.4 (0.124 to 0.676)		.005
Perceived behavioral control	0.497 (0.141 to 0.853)		.007	0.52 (0.244 to 0.796)		<.001
Constant	−1.58 (−2.765 to −0.387)		.01	0.451 (−1.303 to 2.204)		.61

a *R*2 indicates the proportion of variance in the dependent variable (intention to use telemedicine) explained by the independent variables (attitudes, subjective norms, and perceived behavioral control).

b *R*2=0.664.

c *R*2=0.258.

d U-B represents the unstandardized B value, testing whether the coefficient is equal to zero.

e The 95% CI reflects the range within which the true B value is likely to fall.

## Discussion

### Principal Results

A major finding in this study is the low self-reported use of telemedicine among both English and Swedish GPs, even after the COVID-19 pandemic. Practitioners from both countries also reported limited training in telemedicine. Despite this, Swedish GPs demonstrated significantly higher mean scores for intentions to use telemedicine in daily practice compared with their English counterparts. More positive attitudes and greater perceived behavioral control were significantly associated with higher behavioral intention scores among both English and Swedish GPs. In addition, the consistently low levels of reported training in both countries suggest that insufficient training may itself be an important contributor to lower intentions to use telemedicine, underscoring the need for more structured educational efforts.

### Strengths and Limitations

A major strength of this study is the use of a validated, theory-based questionnaire (PAIT), adapted and applied in both Swedish and English primary care contexts. The questionnaire was developed based on the TPB framework and had previously been validated among Swedish GPs, demonstrating strong internal consistency and construct validity. The English version, adapted for the UK health care setting, underwent a translation and back-translation process, ensuring linguistic equivalence and conceptual accuracy. This adaptation enhances the study’s ability to compare findings between English and Swedish GPs while maintaining methodological robustness.

Another strength lies in the use of theory-based survey items that have been previously used in other primary care studies, ensuring that the measured constructs are relevant and reliable. The exclusion of questions related to AI, which had shown limited relevance in the original Swedish survey, further streamlined the questionnaire for the UK primary care context.

However, the study has several limitations. The small English sample limits generalizability, so the findings should be considered exploratory. Future studies should recruit larger samples. One key limitation is the difficulty in estimating the response rate due to the recruitment methods used. As the survey was distributed through various GP networks and professional organizations, the total number of GPs who received the invitation remains unknown. Consequently, it is not possible to draw firm conclusions regarding the generalizability of the results.

Additionally, the sample of English respondents was not fully representative of the national GP population. Of the English participants, 71.2% (37/52) were female, compared with 57% of the GP workforce in England as of May 2023. This overrepresentation of female GPs may have introduced bias in the findings.

Another limitation was the challenge of identifying the specific GP groups that responded to the questionnaire. The distribution method involved contacting GP organizations and networks across southwestern England, with responses collected anonymously. While anonymity is crucial for encouraging honest responses, it also means that duplicate responses could not be detected. Nevertheless, given the time constraints faced by GPs and the voluntary nature of participation, the risk of duplicate responses is believed to be very low.

### Comparison With Prior Work

The low self-reported use of telemedicine in this study is an interesting finding, despite expectations of increased use after the COVID-19 pandemic. One explanation might be that, during the COVID-19 pandemic, practices had a vital role in providing physical contact to organize and manage accurate diagnostics [18] with the purpose of avoiding hospitalization.

Our study shows that Swedish GPs had significantly higher mean scores for predictors and behavioral intentions to use telemedicine compared with English GPs, which might have several explanations. Since 2014, patients in all geographic areas in Sweden have been broadly introduced to video consultations mainly through private health care providers of digital care, leading to substantially increased costs for the regions, raising concerns regarding accessibility, inequity of care, continuity of care, and effective resource use [2]. Today, video consultations, chat-based digital tools, and a national digital contact platform are available across most Swedish regions. Many private digital health care providers have also acquired previously established primary care centers, offering integrated “digi-physical” care.

While the United Kingdom has made significant progress in health care IT, it faces ongoing challenges related to interoperability, adoption, and funding. Although access to telemedicine and digital services is improving, it remains inconsistent across regions, potentially influencing English GPs’ lower intentions to adopt telemedicine. In contrast, Sweden has developed a more integrated health care IT system with higher levels of telemedicine use, prioritizing patient convenience and accessibility.

Other Swedish studies have shown positive attitudes toward telemedicine in primary care, reporting that medical staff seem to appreciate the flexibility of working in a digital context [19], but few consider this a full-time career option [4]. While psychologists report higher intentions to use video consultations compared with physicians and nurses in a similar theory-based survey [15], physicians stress the need for evaluation of telemedicine tools regarding clinical usefulness, patient safety, and involvement of end users in the implementation process [13]. There are few studies discussing English GPs’ intentions to use telemedicine, with similar findings [3,7]. In this study, English GPs responded with low intentions to use telemedicine and little self-reported experience and training on the topic. Telemedicine and its use have been widely debated recently, regarding the practical benefits and setbacks and the optimal methods to achieve adoption [1,20]. Greenhalgh et al [26] discuss the importance of the correct adoption method of telemedicine to remain considerate of both GPs and patients, to maximize the benefits of telemedicine and minimize the setbacks.

Telemedicine appears to have a significant positive effect on many challenges that primary care faces today, such as accessibility or increased patient-centeredness [27]. There is therefore a need for a validated and efficient method of testing GPs’ intentions of using telemedicine to understand predictors of behavioral intentions and allow successful implementation.

### Implications for Research and Practice

Before a more positive attitude toward the use of telemedicine can develop, it is essential to have seamless integration of telemedicine platforms with existing health care systems; comprehensive training for GPs and staff to use telemedicine tools effectively; access to remote diagnostic devices (eg, digital stethoscopes and blood pressure monitors) for patients to use at home; and increased funding for telemedicine infrastructure and services. GPs need to be involved in the development and refinement of telemedicine systems to ensure that the tools meet their needs. A cultural shift is unlikely to occur until skepticism among GPs has been addressed by promoting telemedicine’s ability to enhance accessibility and reduce unnecessary in-person visits.

The method of this study could be used for further studies either in England among English GPs or in other English-speaking countries, or it could be translated and used in other countries to strengthen the knowledge of GPs’ intentions to use telemedicine. This could help better understand the demand and benefits of certain telemedicine adoption methods in either local or global implementation structures and provide a better understanding of simple intentions. Future studies with larger and more representative samples should include training as a formal predictor of behavioral intentions, given its potential relevance for shaping GPs’ intentions to adopt telemedicine.

### Conclusions

This study showed that English GPs reported lower intentions to use telemedicine compared with Swedish GPs. Practitioners from both countries also had little training in telemedicine. The behavioral intentions to use telemedicine among both English and Swedish GPs were mainly influenced by attitudes and perceived behavioral control. The PAIT questionnaire provided valuable insights into similarities and differences between English and Swedish GPs regarding attitudes, behavioral intentions, and self-reported telemedicine use. The PAIT could serve as a useful tool for cross-country comparisons of telemedicine adoption, enabling assessments of attitudes, intentions, and perceived behavioral control across health care systems and jurisdictions. This potential is further enhanced by the recent translation of the PAIT survey into Latvian and Turkish languages by collaborating researchers, broadening its applicability for international studies.

## Supplementary material

10.2196/73609 Multimedia Appendix 1 Survey about physicians’ attitudes and experiences of telemedicine in primary care.

10.2196/73609 Checklist 1 CHERRIES checklist.

## Abbreviations

AI: artificial intelligence
BIT: Behavioural Intentions towards Telemedicine
CHERRIES: Checklist for Reporting Results of Internet E-Surveys
COSMIN: Consensus-based Standards for the Selection of Health Measurement Instruments
GP: general practitioner
PAIT: Physicians' Attitudes and Intentions to use Telemedicine
TPB: theory of planned behavior
