# Supplementary material for: Exploring English and Swedish General Practitioners’ Behavioral Intentions to Use Telemedicine: Comparative Study
Source: JMIR Hum Factors. 2026 Mar 20;13:e73609. doi: 10.2196/73609 (PMC13004589; doi:10.2196/73609)
Supplement: Multimedia Appendix 1 [file humanfactors-v13-e73609-s001.docx]

**Appendix 1**

**Survey about physicians’ attitudes and experiences of telemedicine in primary care**

Telemedicine is a broad term, encompassing different areas. We listed below some examples of telemedicine:

- Digital contacts between patients and care givers (e. g. e-mail, video consultations, chat)
- Monitoring chronic disease with digital tools (mobile applications, remote transfer of vital parameters as pulse, heart rhythm, blood pressure, blood sugar)

This survey has the goal to study your experiences and attitudes towards telemedicine in the three main domains described above: digital contacts or monitoring of chronic disease).

The statements you will read have seven answer options. In most cases, 1 means “Strongly disagree” and 7 means “Strongly agree”, otherwise it is specified with the question.

Choose the alternative that suits you best. There is no right or wrong. There is even place for you to write your opinions in free text, if the presented alternatives do not suit you.

The survey will take you 10 minutes to answer.

Your participation is voluntary, and you can exit the survey at any time. The answers will be collected anonymously and will not be related to you as an individual.

The study is performed by the Center for primary care research in Malmö and Lund University/Exeter University.

Contact information

……………………

1. There is a fast and broad development of digital tools in health care. Do you keep yourself updated?

1 = Not at all 7 = To a large extent

| 1 |  | 2 |  | 3 |  | 4 |  | 5 |  | 6 |  | 7 |  |
| --- | --- | --- | --- | --- | --- | --- | --- | --- | --- | --- | --- | --- | --- |

1. In my everyday work with patients, I use following types of contacts:

1 = Not at all 7 = To a large extent

| Physical consultations |  | 1 |  | 2 |  | 3 |  | 4 |  | 5 |  | 6 |  | 7 |  |
| --- | --- | --- | --- | --- | --- | --- | --- | --- | --- | --- | --- | --- | --- | --- | --- |
|  |  |  |  |  |  |  |  |  |  |  |  |  |  |  |  |
| Telephone consultations |  | 1 |  | 2 |  | 3 |  | 4 |  | 5 |  | 6 |  | 7 |  |
|  |  |  |  |  |  |  |  |  |  |  |  |  |  |  |  |
| Video consultations |  | 1 |  | 2 |  | 3 |  | 4 |  | 5 |  | 6 |  | 7 |  |
|  |  |  |  |  |  |  |  |  |  |  |  |  |  |  |  |
| National e-service portal |  | 1 |  | 2 |  | 3 |  | 4 |  | 5 |  | 6 |  | 7 |  |
|  |  |  |  |  |  |  |  |  |  |  |  |  |  |  |  |
| E-mail |  | 1 |  | 2 |  | 3 |  | 4 |  | 5 |  | 6 |  | 7 |  |
|  |  |  |  |  |  |  |  |  |  |  |  |  |  |  |  |
| Letters |  | 1 |  | 2 |  | 3 |  | 4 |  | 5 |  | 6 |  | 7 |  |
|  |  |  |  |  |  |  |  |  |  |  |  |  |  |  |  |
| Chat |  | 1 |  | 2 |  | 3 |  | 4 |  | 5 |  | 6 |  | 7 |  |
|  |  |  |  |  |  |  |  |  |  |  |  |  |  |  |  |
| SMS |  | 1 |  | 2 |  | 3 |  | 4 |  | 5 |  | 6 |  | 7 |  |

Do you have any free comments about the use of digital contacts?

……………………………………………………………………………………………………………

**We will start with a section including questions about digital contacts in patient care.**

1. The use of digital contacts as e-mail, chat or sms in patient care is:

| Harmful = 1; Beneficial = 7 |  | 1 |  | 2 |  | 3 |  | 4 |  | 5 |  | 6 |  | 7 |  | I don’t know |
| --- | --- | --- | --- | --- | --- | --- | --- | --- | --- | --- | --- | --- | --- | --- | --- | --- |
|  |  |  |  |  |  |  |  |  |  |  |  |  |  |  |  |  |
| Bad = 1; Good = 7 |  | 1 |  | 2 |  | 3 |  | 4 |  | 5 |  | 6 |  | 7 |  | I don’t know |
|  |  |  |  |  |  |  |  |  |  |  |  |  |  |  |  |  |
| Pleasant (for me) = 1; Unpleasant (for me) = 7 |  | 1 |  | 2 |  | 3 |  | 4 |  | 5 |  | 6 |  | 7 |  | I don’t know |
|  |  |  |  |  |  |  |  |  |  |  |  |  |  |  |  |  |
| Worthless= 1; Useful= 7 |  | 1 |  | 2 |  | 3 |  | 4 |  | 5 |  | 6 |  | 7 |  | I don’t know |

Do you have any comments about the use of digital contacts as e-mail, chat or sms?

……………………………………………………………………………………………………………

1. The use of digital contacts as video consultation in patient care is:

| Harmful = 1; Beneficial = 7 |  | 1 |  | 2 |  | 3 |  | 4 |  | 5 |  | 6 |  | 7 |  | I don’t know |
| --- | --- | --- | --- | --- | --- | --- | --- | --- | --- | --- | --- | --- | --- | --- | --- | --- |
|  |  |  |  |  |  |  |  |  |  |  |  |  |  |  |  |  |
| Bad = 1; Good = 7 |  | 1 |  | 2 |  | 3 |  | 4 |  | 5 |  | 6 |  | 7 |  | I don’t know |
|  |  |  |  |  |  |  |  |  |  |  |  |  |  |  |  |  |
| Pleasant (for me) = 1; Unpleasant (for me) = 7 |  | 1 |  | 2 |  | 3 |  | 4 |  | 5 |  | 6 |  | 7 |  | I don’t know |
|  |  |  |  |  |  |  |  |  |  |  |  |  |  |  |  |  |
| Worthless= 1; Useful= 7 |  | 1 |  | 2 |  | 3 |  | 4 |  | 5 |  | 6 |  | 7 |  | I don’t know |

Do you have any comments about the use of digital contacts as video consultations?

……………………………………………………………………………………………………………

1. If the possibility existed, I would use digital contacts in patient care to a larger extent.

1 = Strongly disagree 7 = Strongly agree

| 1 |  | 2 |  | 3 |  | 4 |  | 5 |  | 6 |  | 7 |  | I don’t know |
| --- | --- | --- | --- | --- | --- | --- | --- | --- | --- | --- | --- | --- | --- | --- |

1. For me, using digital contacts in patient care is:

1 = Difficult 7 = Easy

| 1 |  | 2 |  | 3 |  | 4 |  | 5 |  | 6 |  | 7 |  | I don’t know |
| --- | --- | --- | --- | --- | --- | --- | --- | --- | --- | --- | --- | --- | --- | --- |

1. The decision to use more digital contacts is beyond my control.

1 = Strongly disagree 7 = Strongly agree

| 1 |  | 2 |  | 3 |  | 4 |  | 5 |  | 6 |  | 7 |  | I don’t know |
| --- | --- | --- | --- | --- | --- | --- | --- | --- | --- | --- | --- | --- | --- | --- |

1. It is expected of me to use digital contacts in patient care.

1 = Strongly disagree 7 = Strongly agree

| 1 |  | 2 |  | 3 |  | 4 |  | 5 |  | 6 |  | 7 |  | I don’t know |
| --- | --- | --- | --- | --- | --- | --- | --- | --- | --- | --- | --- | --- | --- | --- |

1. I feel under social pressure to use digital contacts in patient care.

1 = Strongly disagree 7 = Strongly agree

| 1 |  | 2 |  | 3 |  | 4 |  | 5 |  | 6 |  | 7 |  | I don’t know |
| --- | --- | --- | --- | --- | --- | --- | --- | --- | --- | --- | --- | --- | --- | --- |

1. I intend to use digital contacts in patient care to a larger extent, if it is clinically adequate for the patients.

1 = Strongly disagree 7 = Strongly agree

| 1 |  | 2 |  | 3 |  | 4 |  | 5 |  | 6 |  | 7 |  | I don’t know |
| --- | --- | --- | --- | --- | --- | --- | --- | --- | --- | --- | --- | --- | --- | --- |

1. I want to use digital contacts in patient care to a larger extent, if it is clinically adequate for the patients.

1 = Strongly disagree 7 = Strongly agree

| 1 |  | 2 |  | 3 |  | 4 |  | 5 |  | 6 |  | 7 |  | I don’t know |
| --- | --- | --- | --- | --- | --- | --- | --- | --- | --- | --- | --- | --- | --- | --- |

Do you have any comments about the use of digital contacts in patient care?

……………………………………………………………………………………………………

**The following section has questions about monitoring chronic disease, as diabetes, with digital tools.**

1. I have experience of using digital tools for monitoring chronic diseases with digital tools (i.e. mobile applications, thumb-ECG):

1 = Not at all 7 = To a large extent

| 1 |  | 2 |  | 3 |  | 4 |  | 5 |  | 6 |  | 7 |  |
| --- | --- | --- | --- | --- | --- | --- | --- | --- | --- | --- | --- | --- | --- |

1. Monitoring chronic disease with digital tools is:

| Harmful = 1; Beneficial = 7 |  | 1 |  | 2 |  | 3 |  | 4 |  | 5 |  | 6 |  | 7 |  | I don’t know |
| --- | --- | --- | --- | --- | --- | --- | --- | --- | --- | --- | --- | --- | --- | --- | --- | --- |
|  |  |  |  |  |  |  |  |  |  |  |  |  |  |  |  |  |
| Bad = 1; Good = 7 |  | 1 |  | 2 |  | 3 |  | 4 |  | 5 |  | 6 |  | 7 |  | I don’t know |
|  |  |  |  |  |  |  |  |  |  |  |  |  |  |  |  |  |
| Pleasant (for me) = 1; Unpleasant (for me) = 7 |  | 1 |  | 2 |  | 3 |  | 4 |  | 5 |  | 6 |  | 7 |  | I don’t know |
|  |  |  |  |  |  |  |  |  |  |  |  |  |  |  |  |  |
| Worthless= 1; Useful= 7 |  | 1 |  | 2 |  | 3 |  | 4 |  | 5 |  | 6 |  | 7 |  | I don’t know |

1. If the possibility existed, I would use digital tools for monitoring chronic diseases, as diabetes, to a larger extent.

1 = Strongly disagree 7 = Strongly agree

| 1 |  | 2 |  | 3 |  | 4 |  | 5 |  | 6 |  | 7 |  | I don’t know |
| --- | --- | --- | --- | --- | --- | --- | --- | --- | --- | --- | --- | --- | --- | --- |

1. For me, using digital tools for monitoring chronic diseases in patient care is:

1 = Difficult 7 = Easy

| 1 |  | 2 |  | 3 |  | 4 |  | 5 |  | 6 |  | 7 |  | I don’t know |
| --- | --- | --- | --- | --- | --- | --- | --- | --- | --- | --- | --- | --- | --- | --- |

1. The decision to use more digital tools for monitoring chronic diseases is beyond my control.

1 = Strongly disagree 7 = Strongly agree

| 1 |  | 2 |  | 3 |  | 4 |  | 5 |  | 6 |  | 7 |  | I don’t know |
| --- | --- | --- | --- | --- | --- | --- | --- | --- | --- | --- | --- | --- | --- | --- |

1. It is expected of me to use digital tools for monitoring chronic diseases in patient care.

1 = Strongly disagree 7 = Strongly agree

| 1 |  | 2 |  | 3 |  | 4 |  | 5 |  | 6 |  | 7 |  | I don’t know |
| --- | --- | --- | --- | --- | --- | --- | --- | --- | --- | --- | --- | --- | --- | --- |

1. I feel under social pressure to use digital tools for monitoring chronic diseases in patient care.

1 = Strongly disagree 7 = Strongly agree

| 1 |  | 2 |  | 3 |  | 4 |  | 5 |  | 6 |  | 7 |  | I don’t know |
| --- | --- | --- | --- | --- | --- | --- | --- | --- | --- | --- | --- | --- | --- | --- |

1. I intend to use digital tools for monitoring chronic diseases in patient care to a larger extent, if it is clinically adequate for the patients.

1 = Strongly disagree 7 = Strongly agree

| 1 |  | 2 |  | 3 |  | 4 |  | 5 |  | 6 |  | 7 |  | I don’t know |
| --- | --- | --- | --- | --- | --- | --- | --- | --- | --- | --- | --- | --- | --- | --- |

1. I want to use digital tools for monitoring chronic diseases in patient care to a larger extent, if it is clinically adequate for the patients.

1 = Strongly disagree 7 = Strongly agree

| 1 |  | 2 |  | 3 |  | 4 |  | 5 |  | 6 |  | 7 |  | I don’t know |
| --- | --- | --- | --- | --- | --- | --- | --- | --- | --- | --- | --- | --- | --- | --- |

Do you have any comments about the use of digital tools for monitoring chronic diseases in patient care?

**This section has general questions about you.**

Are you:

| Man |  | Woman |  | This classification does not suit me | | | |
| --- | --- | --- | --- | --- | --- | --- | --- |
|  |  |  |  |  |  |  |  |
|  |  |  |  |  | | | |

How old are you?

…………………………

Are you:

| Specialist in family medicine | Resident in family medicine | Intern / Other assistant physician | Other specialty |
| --- | --- | --- | --- |

How long have you worked in primary care (number of years)?

………………………………

Do you work in public or private care?

| Public care | Private care |
| --- | --- |

I do need more information, knowledge and training of using digital tools in primary care.

1 = Strongly disagree 7 = Strongly agree

| 1 |  | 2 |  | 3 |  | 4 |  | 5 |  | 6 |  | 7 |  | I don’t know |
| --- | --- | --- | --- | --- | --- | --- | --- | --- | --- | --- | --- | --- | --- | --- |

Do you have any other comments?

………………………………………
