# Supplementary material for: Exploring English and Swedish General Practitioners’ Behavioral Intentions to Use Telemedicine: Comparative Study
Source: JMIR Hum Factors. 2026 Mar 20;13:e73609. doi: 10.2196/73609 (PMC13004589; doi:10.2196/73609)
Supplement: Checklist 1 [file humanfactors-v13-e73609-s002.docx]

Checklist for Reporting Results of Internet E-Surveys (CHERRIES)

| **Item category** | **Check-list item** | **Explanation** |
| --- | --- | --- |
| **Design** | Survey design | Web-based survey to physicians in primary care from two counties in Southern Sweden (N=820)  between March-May 2022 and England (number of invitation recipients unknown because of the variety of recruitment channels used) between March-May 2023. |
| **IRB (Institutional Review Board) approval and informed consent process** | Ethical approval | The Swedish Ethical Review Authority considered that ethical approval and participant written consent was not necessary for this type of study using anonymous data. Ethical approval was granted by the review board at Exeter University in March 2023 (Ethics Application ID: 845606) |
|  | Informed consent | An informed consent was collected at the start of the survey, containing a clear statement about the length of the survey, who conducted the study, the purpose of the survey and that answering was voluntary. |
|  | Data protection | In Sweden, data was collected with REDCap and in England with Microsoft Forms, secure web applications for building and managing online surveys and databases. All answers were anonymous. |
| **Development and pre-testing** | Development and testing | The survey was tested for construct validity and reliability in Sweden and later adjusted for English conditions with some minor language corrections. Internal validity was psychometric tested by five Swedish physicians with good understanding of the topic, evaluating if the questionnaire was capturing the research question. They made written notes on paper questionnaires regarding leading or confusing questions. The comments of the expert group lead to minor changes in wording and formatting.  Then we performed a pilot study at two PHCCs by sending the questionnaire to 24 physicians. Internal consistency was tested with Cronbach’s α (CA). Items with low CA (<0.6) were removed from the questionnaire.  Test-retest reliability was studied by sending the questionnaire a second time to the same respondents after two weeks. They were asked to fill in initials in “free answer” box in order to identify unique answers. Twelve physicians returned the questionnaire.  The Pearson correlation coefficient was measured, defining a value of >0.6 as acceptable. |
| Recruitment process and description of the sample having access to the survey |  | All physicians at the PHCCs in Southern Sweden were invited to participate in the survey by e-mailing an electronic link.  In England, GPs were invited to participate in the survey by e-mailing an electronic link and through newsletters and GP magazines. |
| Survey administration | Survey type | The survey was sent as an “open survey”. |
|  | Contact mode | In Sweden, the web-link was sent to the PHCC managers together with an information letter for further dissemination to physicians, allowing for web-based data entry. In England, the survey was sent through social media (Twitter, Facebook), as well as through the Royal College of General Practitioners, the British Medical Journal, GP magazine, Pulse magazine, Local Medical Committees, ‘GP online’, ‘eLearning’, ‘GP survival’ and Somerset GP Education. |
|  | Advertising the survey | In Sweden, the main investigators informed primary care physicians at local conferences about the survey. I England, the advertising was made through the channels described above. |
| Survey administration | Web-based link | The survey was sent as an e-mail link to a secured web-page managed by REDCap or Microsoft Forms with automatic capturing of the responses. Completing the questionnaire was optional. No incentives were offered. |
|  | Timeframe | The survey ran from March to May 2022 (in Sweden) and March-May 2023 (in England). Two reminders were sent by e-mail during the data collection. |
|  | Completeness check | The respondent could submit the survey without fully completing the survey. It was not technically possible to do consistency or completeness checks before the questionnaire was submitted. |
|  | Randomisation | Items were not randomised. |
|  | Adaptive questioning | All surveys had the same questions. |
|  | Number of questions/items | 26 |
|  | Number of sub-items | 44 |
|  | Number of screens | 9 |
|  | Completeness check | Completing all questions was not mandatory. |
|  | Review check | Respondents were able to review and change the answers with a “back” button. |
| Response rates | Unique site visitor | We did not determine if the respondents were unique site visitors |
|  | Completion rate | Not able to assess due to the distribution methods through the managers |
| Preventing multiple entries from the same individual | Cookies, IP-check, log-file analysis or registration | No cookies, IP-check, log-file analysis or registration were used, to avoid multiple entries from the same individual. |
| Analysis | Handling of incomplete questionnaires | If more than half of the questions for each item were not answered, the overall score was defined as missing. The other items with at least half of the questions answered were used for analysis. |
|  | Timestamp | We did not measure a cut-off point for the time needed to deliver the completed surveys. |
|  | Statistical correction | No correctional analysis was made. |
